# Supplementary material for: Are carbon emissions trading and green financial instruments synergistic? -Comprehensive quantitative research based on content analysis
Source: PLoS One. 2024 Mar 7;19(3):e0298601. doi: 10.1371/journal.pone.0298601 (PMC10919601; doi:10.1371/journal.pone.0298601)
Supplement: S1 Data — (ZIP) [file pone.0298601.s001.zip › Data/地级市Z_F工作报告.zip!/║■▒▒/╛ú├┼╩╨/└┤╫╘╣1⁄2╓┌║┼í╛┬φ┐╦╩2╛▌═°í┐_2014.docx]

各位代表：

　　现在，我代表市人民政府向大会报告工作，请予审议，并请各位政协委员和列席人员提出意见。

　　一、2013年工作回顾

　　刚刚过去的一年，面对错综复杂的宏观经济环境，面对日趋激烈的区域竞争压力，面对不断增多的社会管理难题，我们在省委、省政府和市委的坚强领导下，与全市人民一道，团结拼搏、砥砺奋进，较好地完成了八届人大二次会议确定的目标任务。全年实现地区生产总值1202.61亿元，增长10.5%；规模以上工业增加值655.75亿元，增长13%，总量居全省第4位；全社会固定资产投资1014.64亿元，增长27.7%；社会消费品零售总额395.48亿元，增长14%；居民消费价格涨幅为3.2%；地方公共财政预算收入59.84亿元，增长18.1%；实际外商直接投资26275万美元，增长21.3%；出口61752万美元，增长32.6%；城镇居民人均可支配收入19820元，增长12.1%；农民人均纯收入10615元，增长13.1%，总量居全省第2位；城镇登记失业率控制在4.1%以内；人口自然增长率4.2％。；超额完成省定节能减排目标。

　　（一）“两大战略”全面实施。中国农谷三大示范区规划体系基本建立。核心区农谷大道全面通车，组建了中国农谷联合产权交易中心和中国农谷农科院，举办了首届中国农谷论坛，联想控股、中兴集团、湖北联投、东风井关等知名企业相继落户。“荆三角”赵集智慧农业园、现代林业精品示范园、双碑现代农业循环园发展势头良好。杨竹流域双龙苑万人社区、皮集新社区开工建设。柴湖振兴发展工作全面启动，15项规划基本完成，省级荆门大柴湖经济开发区获批筹建，柴湖工业园一期建设进展顺利，建成四新村“四网千棚”花果蔬示范基地。

　　（二）项目投资有效扩大。深入开展项目建设百日大会战和招商引资大竞赛，精心组织首届楚商大会荆门分会场等招商活动，项目落地明显加快，全市在建亿元以上项目471个，竣工投产项目110个。万达广场、红星美凯龙、联邦航太、襄荆高速公司总部、麒麟集团万华城等项目落户荆门，两岸三地华中创业中心、京峻汽车铸件、盈德煤制氢、腾宝建材等项目开工建设，金泉新材料、格林美东区、洪森食品工业园、楚商国际产业城、华能热电联产等项目加快推进，百盟慧谷一期、戴蒙德新厂、明达玻璃一期、捷力通汽车零部件、众诚冷链物流、天茂20万吨碳四、五三柒零纺织一期等项目竣工投产。蒙华铁路（荆门段）前期工作基本完成，荆门石化油品质量升级及适应性改造获批开展前期工作。

　　（三）工业经济逆势而进。出台《关于促进工业经济平稳较快发展的意见》，落实企业控息单、缓税单和退城进园单，市财政安排3000万元支持企业发展。实施“双千亿产业计划”，农产品加工产值率先突破1000亿，化工产值达到897亿元，中国农谷农副产品加工、京山轻工包装机械、钟祥农产品加工和东宝磷化工等产业集群纳入省重点扶持行列。全市新增亿元企业68家、规模企业140家，分别达到423家、922家。省级工程中心、研发中心和重点实验室达到16家，新增高新技术企业20家，高新技术产业增加值达到114亿元。国家循环经济示范市创建通过省级考核验收，格林美城市矿产资源循环产业园被纳入国家示范基地。“一核六片十五园”完成工业增加值370亿元，增长15%，荆门高新区和东宝工业园分别居省级开发区综合考评、竞进提质竞赛第一名，京山经济开发区升格为省级高新区。农产品、化工医药产品出口增势强劲，楚玉食品、大峪口化工出口均突破5000万美元。京山轻机印度工厂正式投产运营。钟祥和东宝连续六年跻身全省县域经济20强。

　　（四）现代农业稳步发展。坚持“抗大旱，保生活、保生产、保发展”，战胜“四年连旱”灾害。调整农业种植结构27万亩，粮食增产1.09亿斤，实现“十连增”。水产品产量增幅连续三年居全省首位。畜牧业产值达到110亿元。深入实施“四个一批”工程，新增10亿元龙头企业3家、50亿元企业1家。沙洋农产品加工园区被评为全国示范园区，京山、钟祥农产品加工园区被评为全省先进园区。主要农作物耕种收综合机械化水平达到71%，五洋等4个农机专业合作社被评为全国示范社。新型农业经营主体达到1.5万家。新增土地流转面积56.3万亩，转移农民12万人。实施土地整治规模70万亩，新增城乡增减挂钩指标3000亩。硬化渠道880公里，扩挖塘堰10472口，完成100座水库除险加固工程，解决农村10万人饮水安全问题，我市被评为全国节水型社会建设示范区。完成植树造林17.45万亩，“四旁”植树1083万株。钟祥、沙洋连续三年被评为全省“三农”发展先进县（市）。

　　（五）第三产业不断壮大。沙洋油菜花海名列中国美丽田园油菜景观第二位，彭墩乡村世界跻身国家4A级景区，绿林山景区晋升为省级森林公园，马河青林寨景区投入运营。全年实现旅游总收入90.61亿元，增长20.3%。成功举办“舌尖上的中国农谷”武汉展销会及推介月活动。建成人民万福国际商贸城，荆门大家装、湖北供销民生广场、中国农谷荆门农产品物流中心等项目扎实推进。净增限上法人企业251家。新建、改造村级综合服务社300家，完成中心城区菜市场标准化改造5家。实现电话银行村级全覆盖、村镇银行县域全覆盖，设立小额贷款公司16家，发行二期城投债16亿元，中小企业金融服务网正式运营。全市金融机构各项贷款余额560.42亿元，比年初新增123.08亿元。新洋丰肥业借壳上市获中国证监会核准，京大饲料成为全市首家新四板上市企业。家政、物业、保险、中介等业态进一步发展。

　　（六）城乡建设统筹推进。全市城镇化率达到51.2%。编制完成新一轮荆门城市总体规划，漳河新区、荆门高新区、荆门化工循环产业园、东宝工业园“四大平台”规划体系逐步完善，“数字荆门”地理空间框架投入试运行。漳河新区道路骨架正在拉开，政务中心、凤凰湿地公园一期等工程稳步实施。有序推进老城区改造，积极创建花园城市，我市获得2016年第十二届中国菊花展举办权。全面实施城市精细化管理，推行“马路局长”工作法，数字化城市管理系统通过国家住建部考核验收。中心城区违法建设得到有效控制，公路治超初见成效。新增空调公交车100辆。县（市、区）城市新区建设加快推进，4个镇域城市试点逐步深化，官垱镇被列为省“四化同步”第一批试点示范乡镇。完成新型农村社区布点规划和首批100个新型农村社区详细规划，在建新型农村社区93个。深入开展“万名干部进万村洁万家”活动，村容村貌逐步改观。国家环境保护模范城市、国家卫生城市创建工作扎实推进，漳河水库被纳入国家良好湖泊生态环境保护试点,京山成为全省唯一的省级生态县。西外环等5条一级公路、东桥至义和旅游环线等8条二级公路开工建设，建成通乡公路54.3公里、通村沥青（水泥）路908公里。

　　（七）人民生活持续改善。市本级民生和各项公共事业投入22亿元，占公共财政支出总额的73.1%，高于全省2.7个百分点，2013年初确定的民生实事全部兑现。新增城镇就业51598人，扶持创业6449人、带动就业25687人。市创业小额贷款担保中心投入运行。五大社会保险参保190.8万人次，城乡居民养老保险参保率达99.9%。启动基本医疗保险普通门诊统筹，实施城乡居民大病保险，取消城镇医保报销封顶线。深化县级公立医院改革，钟祥市人民医院“先看病，后交钱”服务模式在全省推广。新农合参合率99.9%，政策范围内补偿率76.2%。整合优化市直医疗卫生资源，市妇幼保健院改扩建项目一期工程投入运营。荆门市中医医院、钟祥市中医院成功创三甲。新建、改造乡镇卫生院面积21.3万平方米。开展中国侨联“健康光明行”白内障免费复明活动。新开工建设保障性住房、棚户区改造住房14203套，基本建成11834套，分配入住7744套。城乡低保再次提标，发放医疗救助金1860万元、临时救助金1855万元。全市养老床位达到8367张。落实义务教育经费保障机制改革资金2.42亿元，筹集3567万元资助各级各类学校学生36400人。钟祥、东宝义务教育均衡发展通过国家认定，我市荣获“中国教育创新奖”。新建、改扩建30所公办幼儿园,掇刀名泉小学建成投入使用。湖北信息工程学校被评为全国首批职业教育改革与发展示范学校，市高级技工学校申建为荆门技师学院。荆楚理工学院职业技术学院挂牌成立。圆满承办国际网联U18青少年网球赛、全国U18女子足球赛和第五届漳河游泳节，电影《农谷之恋》纳入全国公益电影放映目录。建成数字农家书屋289家。我市荣获第七届“全国科技进步考核先进城市”。夺得省党政领导线人口计生目标管理责任制考评一等奖。完成“六五”普法中期目标，办理各类法律援助案件、事项1.1万件。平安荆门建设扎实推进，影响群众安全感的暴力犯罪和可防性案件明显下降，社会大局保持稳定。

　　（八）自身建设得到加强。认真贯彻中央八项规定、省委六条意见和市委实施细则，切实改进工作作风。坚持依法行政，155件人大代表建议、418件政协提案全部办结。行政首长出庭应诉率达98.5%。完善市政府常务会议等议事规则和决策程序。启动事业单位分类改革。深化行政审批改革，免收行政审批服务费5100万元，市级行政审批事项缩减至126项，行政审批再提速12%。强化公共资源交易综合监管，交易总额达到120亿元。扎实开展“打开荆门、大开荆门”大讨论活动和“效能风暴”行动，举办两场电视问政，进行明察暗访320次，发出电子监察黄牌预警信息35起，问责追查案件103个。土地执法名列全省效能考核第一。加强审计监督、行政监察，有效开展行政权力清理和确认，严肃查处各类违纪违法案件，廉政建设和反腐败工作取得新成绩。

　　进一步密切与工会、共青团、妇联等人民团体的联系，关工委、老龄、残疾人、慈善等事业进一步发展，人民防空、优抚安置、拥军优属和军民共建扎实推进。援恩、援疆工作成效明显，扶贫开发、移民、民族宗教、外事、侨务、统计、物价、气象、防震减灾、住房公积金、新闻出版、档案、地方志、机关事务管理、保密等工作取得新进展。

　　各位代表，刚刚过去的一年，我们举办了隆重而节俭的建市30周年纪念活动，形成了“三十而立、只争朝夕”的强大发展气场；我们合力共为，推动柴湖振兴发展上升为省级战略，使荆门成为全省唯一实施两个省级战略的市州；我们抢抓机遇，申报成为湖北唯一的国家节能减排财政政策综合示范城市，被纳入国家老工业基地改造规划，荆门高新区成功晋升为国家高新区。所有这些都为我市改革发展打下了坚实的基础，提升了荆门在全省乃至全国发展大局中的地位。成绩来之不易，这是省委、省政府和市委正确领导的结果，是市人大、市政协监督和支持的结果，是全市人民齐心协力、顽强拼搏的结果。在此，我代表市人民政府，向全市人民，向人大代表、政协委员，向各民主党派、工商联、无党派人士和人民团体，向驻荆部队、武警官兵、政法干警和中省垂管单位，向所有关心、支持荆门发展的各界朋友和荆门籍在外人士，表示衷心的感谢并致以崇高的敬意!

　　在总结成绩的同时，也应清醒地看到，我市经济社会发展仍存在一些突出问题。主要是：经济总量不够大，重大项目偏少，财政收支矛盾突出，经济回升向好的基础还不牢固；产业结构不尽合理，环境容量不足，转型发展面临较大压力；社会事业发展与人民群众的新期待还有一定差距，维护社会和谐稳定的任务还很艰巨；少数部门和干部思想不够解放、工作作风不实、执行落实不力、办事效率不高的问题依然突出。对此，我们将采取有效措施加以解决。

　　二、2014年工作安排

　　2014年，是贯彻党的十八届三中全会精神、全面深化各项改革的第一年，是深入实施“两大战略”的关键之年，也是实施“十二五”规划的攻坚之年。虽然世界经济新的增长动力源尚不明朗，国内经济运行存在下行压力，但更要看到，随着我国全面深化各项改革的红利不断释放，随着全省“建成支点、走在前列”和“两圈两带”战略的全面实施，随着中国农谷建设和柴湖振兴发展的深入推进，我市进入了一个新的发展阶段。我们一定要进一步解放思想，增强进取意识、机遇意识、责任意识，坚持底线思维，搞好统筹兼顾，奋力开创荆门科学发展、跨越式发展的崭新局面。

　　今年政府工作的总体要求是：全面贯彻落实党的十八大和十八届二中、三中全会精神，坚持“稳中求进、改革创新”的总基调，贯彻“竞进提质、升级增效”的总要求，按照“加速转型、缩差领跑”的基本思路，深入实施中国农谷和柴湖振兴发展“两大战略”，着力兴财力增强之政、抓城乡统筹之政、务民生改善之政、行廉洁务实之政，加快建设“四个荆门”，为顺利完成“十二五”规划目标任务和提前全面建成小康社会奠定坚实基础。

　　今年经济社会发展主要预期目标是：地区生产总值增长11%，规模以上工业增加值增长15%，全社会固定资产投资增长25%，社会消费品零售总额增长14%，居民消费价格涨幅控制在4.0%以内，地方公共财政预算收入增长13%，出口总额增长12%，实际外商直接投资增长12%，城镇居民人均可支配收入增长11%，农民人均纯收入增长12%，城镇登记失业率控制在4.5%以内，人口自然增长率控制在5.5％。以内，节能减排完成省定目标。

　　重点抓好以下工作：

　　（一）着力推动转型升级，夯实工业主导地位。

　　调整优化产业结构。坚持工业强市、产业富民，在做大总量中推进转型发展。以荆门石化1000万吨技改扩能、油品升级、节能环保工程建设为契机，推动石化产业链向横向拓展和下游延伸。加强中低品位磷矿石综合利用，积极开发缓控释肥、生物化肥等新型肥料，鼓励开发硫酸钙晶须、工业级磷酸一铵等精细磷化工产品。支持食品加工行业加大联合、兼并和重组力度，增创发展新优势。加快推进葛洲坝水泥异地技改，推广沙洋玻璃行业转型发展经验，提升建材产业发展水平。抢抓国家有序放开民用航空、通用航空机遇，实施漳河机场改扩建工程，抓好水陆两栖飞机、浮空器研发等项目，建设荆门航空科技产业园，打造华中地区最具竞争力的航空产业基地。积极发展生物质发电，支持沙洋新能源汽车产业发展。围绕创建国家循环经济示范城市，做大做强三大特色循环产业园，组织实施50个重点循环经济项目，建设国家“城市矿产”回收体系创新基地。力争化工产业总产值超过1000亿元，建材、机电、轻纺等产业产值均突破400亿元，新兴产业产值达到200亿元。

　　加快园区提档升级。深入推进“一核六片十五园”建设，实施“项目满园”工程，进一步提高投资强度和产出效益。支持荆门高新区按照“要素高地、产业新区、科技新城”定位，用好国家高新区、中国光谷荆门产业园、生物医药产业基地等名片，招大商、招真商；发挥格林美、福耀、中集、李宁等骨干企业作用，抓续建、扩产能，力争项目建设到位资金超过100亿元，规模工业总产值增速、高新技术产值占比均超过40%。支持荆门化工循环产业园完善配套功能，推进碳四、碳一、芳烃三大产业链项目建设。支持东宝工业园培育发展电子、装备和机械制造、森工家具产业集群，打造千亿园区。支持各县（市、区）以开发区（高新区）、乡镇工业集中区为支撑，突破性发展县域工业，实现县域经济“保强进位”。

　　培育壮大市场主体。鼓励新洋丰、格林美、葛洲坝水泥等企业向百亿企业迈进，力争华能、金鹰能源等企业产值突破10亿元。实施中小企业成长工程，新增规模工业企业130家。建立金融、信息、技术、市场服务体系，实施回归工程，对本土创业企业与招商引资企业同等对待，力争市场主体达到13.5万个。深入实施质量兴市、名牌兴市战略，扩大名牌产品生产规模和市场占有率。强化资金、用地、用工等要素保障，做好重点企业、重点行业的跟踪服务。

　　推进创新驱动发展。深入实施“龙泉英才”计划，引进能够支撑产业发展的领军人才与创新团队。做大做强院士工作站品牌。抓好百家重点企业技改示范和创新示范，引导和支持企业参与各类产学研对接活动，打造产业技术创新联盟，力争新增省级以上高新技术企业25家，高新技术产业增加值占地区生产总值比重达到9.5%。实施工业化与信息化融合示范工程，新增1-2家企业纳入国家试点示范。

　　（二）着力发展现代农业，促进农业农村稳定发展、农民持续增收。

　　发展现代高效农业。推进国家现代农业示范区建设，提高农业综合生产能力。实施百万亩农业高效丰产工程，建设250万亩优质水稻、160万亩优质油菜、汉江流域40万亩优质棉花板块基地，力争粮食增产1亿斤。创建全省现代畜牧业示范区，抓好钟祥牧原200万头生猪等畜禽养殖项目建设。发展现代渔业，推广“香稻嘉鱼”等稻田综合种养模式50万亩，打造特色水产。建设城郊设施蔬菜基地和汉江沿岸蔬菜板块基地，确保“菜篮子”产品有效供给。深化“四个一批”工程，力争国宝、洪森产值突破百亿元,宝源、广源、兴农产值分别超过50亿元，农产品加工产值与农业总产值之比达到3：1。推广立体种养、秸秆养畜、病虫害绿色防控等新技术新模式，加大滴灌喷灌、地膜覆盖等节水技术应用力度，发展生态友好农业。推行农产品原产地可追溯制度和产地准出制度，力争农业“三品一标”质量抽检合格率达到98%以上。

　　加快中国农谷示范区和大柴湖经济开发区建设。在中国农谷核心区，启动国家农业科技园区创建工作，加快推进奈美生态小镇、联想高档水果、汇源生态产业园等项目，建成湖北文化创作交流基地一期、院士村一期及中国农谷农科院等项目。在“荆三角”先行区，加强五洋线、荆新线、207国道沿线城镇带和景观带建设，实施国家级农产品加工园、菜籽油交易中心、农村信息化样板区等项目。在杨竹流域示范区，抓好彭墩“1+5”、双龙苑等新型社区建设，打造生态观光走廊。在大柴湖经济开发区，实施倒口电排站更新改造、东西干渠整治等工程，加大农村危房和棚户区改造力度，争取国家避险解困试点通过省、中央评审。加强柴湖工业园区和现代物流园基础设施建设。以四新村花果蔬基地为示范，实行整村土地流转，培育壮大特色板块基地。抓好感恩自强文明宣传教育活动，推进“柴湖”变“财湖”。

　　健全“三农”服务体系。支持种子工程、丰产栽培、疫病防控、防灾减灾等领域的科技创新和推广，加强气象灾害信息预警预报。建设全国一流农用航空站。扩大油菜机收秸秆粉碎还田面积，力争主要农作物耕种收综合机械化水平达到73%。完善“以钱养事”机制，推行合作式、订单式、托管式等服务模式。在土地流转集中地建立中心粮仓，创新小农水管护机制。

　　努力增加农民收入。稳步发展休闲农业、观光农业，引导富余劳动力就近就业、外出务工和自主创业，拓宽农民增收渠道。按期足额发放各项农业直补资金，确保农民政策性增收。发展壮大村级集体经济，加强农村集体资金、资产、资源管理。做好移民工作，促进老区和贫困地区发展，努力实现同步全面小康目标。

　　（三）着力培植发展后劲，提高经济增长质量和效益。

　　全力推进重大项目建设。全年计划开工建设亿元以上项目450个，完成投资650亿元。做好中石化油品质量升级及适应性改造、碾盘山水利水电枢纽、屈家岭遗址公园、荆门通用航空产业基地、荆门热电厂四期等40个项目前期工作，加快华工科技产业园、中兴循环产业园、万达广场、红星美凯龙、盈德煤制氢等50个重点项目建设，力争格林美东区、明达超薄玻璃技改、台湾宝成工业园、华能热电联产、渝楚化工等项目建成投产。

　　加快培育消费新增长点。推动明显陵、绿林山及漳河等景区提档升级，力争全市旅游总收入达到106亿元以上。规划建设漳河新区中央商务区、总部经济区和动漫文化创意园，推进荆门组合港石牌港区、沙洋港区建设，实施杨家桥物流中心、彭墩汉光农业物流园和各县（市、区）物流园区等项目。加速商贸等生活性服务业提档升级，推行农超、农批、农校、农餐对接新模式，高标准建设2-3条特色美食街。组建供销集团，打造本土百亿商业航母。扩大文化旅游、家政养老、休闲健身、节能环保产品等消费，发展电子商务和网络购物等新型消费业态。培育发展移动多媒体、物联网和公共无线上网等信息产业，推进智慧城市建设。

　　进一步扩大对内对外开放。坚持招商选资、绿色引资，重点引进税收贡献大、能够优化荆门产业结构的项目。深化与央企的战略合作。积极参与第九届中博会等重大招商活动。强化招商项目后续服务和巡回督查，确保项目落实到位。坚持非禁即入，鼓励更多的民间资本进入市政、金融、能源、教育、文化、医疗等领域。主动融入全省“两圈两带”战略，加强同周边城市的横向经济联合与协作。扩大外派劳务人员规模，引导有实力企业到境外发展。做大做强外贸主体，进一步拓展国际市场。

　　（四）着力打造美丽荆门，推进城乡发展一体化。

　　加快建设鄂中城市群。坚持城乡互化发展，让城市融入大自然，让居民望得见蓝天白云、看得见青山绿水、呼吸得到新鲜空气。编制鄂中城市群规划，健全市域城镇体系规划。围绕创建国家绿色生态示范城区，加快漳河新区建设。重点抓好政务中心及配套设施建设，拉通漳河大道城区段，实施双喜大道、荆山大道、天鹅路等道路工程，构建“四横六纵”道路骨架；改造惠民水果市场片区、龙山脊片区和白云西片区；建设双仙河等水网生态工程，建成凤凰湿地公园一期；新建市民广场和荆西公园；整体推进漳河集镇改造，打造北欧峡湾风情小镇。加强中心城区河道卫生、排污、绿化及设施管理，开展城市地下管线现状普查，完善泉口路、象山大道、长宁大道等路段防洪排水设施。启动中国人居环境奖创建活动，积极迎接国家园林城市复检和省“楚天杯”考核。支持钟祥莫愁湖生态新区、京山温泉新区、沙洋滨江新区和东宝北城新区、掇刀长坂新区、荆门高新区观堰湖生态科技新城建设，促进县市城区扩容提质。培育镇域城市、中心镇和特色镇，办好官垱“四化同步”省级示范镇试点，抓好孙桥等5个省级新农村试点镇和42个省级新农村示范村建设。

　　综合整治城市环境。深入实施城市管理三年行动计划，提高精细化管理水平。健全市、区、街办、社区四级联动的城管体制，延伸城市管理考评。打造2-3条省级市容环境美好示范路。优先发展公共交通，增加公共停车泊位，新建海慧路——象山二路、海慧体育场、天鹅路——团结街3处微循环，着力缓解交通拥堵问题。坚决遏制违法建设，分类处置存量违建30%以上。推进高速公路、国（省）道和景区公路沿线环境治理。

　　加大生态环境保护力度。深化国家环保模范城市和森林城市创建，推进国家节能减排财政政策综合示范城市建设。编制中心城区生态环境保护规划，严厉禁止劈山建房行为，构建绿色屏障。落实耕地保护共同责任机制，推进集约节约用地。实行最严格水资源管理制度，实施漳河保护“三圈”规划，加强湿地保护。建设化工循环产业园污水处理厂。综合整治荆门城郊采石场、城市扬尘、餐饮业油烟、秸秆焚烧等突出环境问题。加强地质灾害防治，抓好矿产采空区生态环境恢复治理。集中回收处理废纸、废旧电子产品，综合利用工业固体废弃物。支持京山创建国家生态县、国家生态文明先行示范区。

　　着力建设美丽乡村。完成第二批100个新型农村社区详细规划，统筹推进中心城区、县市城区和重点镇周边、中国农谷示范区的新型社区建设。探索“政府+公司+农户”农村环境污染治理模式，创建生态镇、村。开展“绿满荆门”行动，完成植树造林15万亩，建设绿色通道500公里。组织开展“美丽村庄”评选活动。

　　加强城乡基础设施建设。抓好双喜大道东、西隧道的规划建设，全线开工建设东西外环，构建中心城区“三环八射六隧”交通格局。成立项目公司，多方筹措资金，力争我市开通动车组前期工作取得实质性进展。开工建设蒙华铁路（荆门段）、枣潜高速公路（荆门段）、钟祥汉江二桥等项目。建成荆新线沙洋至潜江段等一级公路，建设、改造二级公路122公里、通乡公路60公里、通村沥青（水泥）路300公里。加快中心城区供热管网建设。实施汉江堤防加固工程，加快汉江以东引水项目建设，完成56座重点小（二）型病险水库除险加固任务，抓好塘堰、小型水库清淤改造扩挖，疏浚渠道1000公里，硬化末级渠道500公里。

　　（五）着力推进重点领域改革，增强发展内生动力。

　　推进行政体制改革。深化卫生、计生机构改革，抓好工商、质监管理体制改革，完成食品药品监管体制改革。合理调整市、区事权和财权，赋予镇域城市和重点镇相应管理权限。加快事业单位分类改革，推动市艺术剧院等一批经营类事业单位转企改制。抓好公共资源交易改革，构筑统一规范的阳光交易平台。推行政府购买服务，支持社会组织进入群众生产生活性服务领域。开展专项整治，规范中介服务机构行为。

　　深化企业改革。完善现代企业制度，探索建立以管理层和骨干员工为主体的员工持股机制。支持金龙泉等市属国有企业引进战略合作者，发展混合所有制经济。筹建粮食集团，完成城建集团所属企业公司制改革，增强交通投资公司市场投融资能力。坚持以管资本为主，完善国有资产监管体制，提高国有资本收益上交公共财政的比例。

　　加快投融资体制改革。鼓励和支持新洋丰、凯龙、宝源等企业在资本市场融资，力争1-2家企业在主板上市，5-10家企业在新三板挂牌，15-20家企业在武汉股权托管交易中心挂牌。引进1-2家股份制商业银行在我市设立分行，新增5家小额贷款公司，支持证券业、保险业在我市设立分支机构。调动银行业金融机构积极性，新增信贷投放100亿元。严厉打击恶意逃废金融债等行为，规范民间借贷，优化金融生态环境。做好城投债发行工作。抓好“营改增”试点。强化财源建设，建立项目优选机制，推进财政专项资金竞争性分配改革。

　　创新农业经营体系。实施土地整治项目50万亩、增减挂钩项目1万亩，创建全国全域土地整治和城乡建设用地增减挂钩示范市。推进“两转三同步四集中”，加大土地整村、整镇流转力度，在中国农谷核心区、“荆三角”掇刀区域、杨竹流域东宝区域逐步实现全域土地流转、全程机械作业、全面绿色种养。引导工商资本到农村发展现代种养业，培育壮大家庭农场、专业大户、农民专业合作社、龙头企业和农民经纪人队伍，发展适度规模经营，力争新型农业经营主体突破2万家。抓好农村集体建设用地和宅基地使用权确权登记，稳妥开展农村集体建设用地和农民住房抵押、担保、转让试点，赋予农民更多财产权利。

　　（六）着力保障和改善民生，增进人民福祉。

　　提升民生保障水平。坚持稳定岗位与扩大就业并重，严格落实一系列积极就业政策，促进农村劳动力转移就业和城镇困难人员就业，做好以高校毕业生为重点的青年创业就业工作。鼓励企业建立职工之家，构建和谐的劳动关系。推进社保由制度全覆盖向人口全覆盖转变，力争新增五大社会保险参保2.8万人次。加强社保基金管理，确保按时足额落实各类社保待遇。做好失地农民的社会保障工作。稳步推进城镇基本服务常住人口全覆盖，保障进城农民工合法权益。完善城乡低保、医疗救助和临时救助制度，关注失独家庭群体，提高农村五保供养水平。健全城乡养老服务体系，加强老年人活动场所建设，力争失能半失能老人托养中心投入运营。抓好保障性住房建设和供给，做好棚户区改造工作，促进房地产市场健康发展。全面推进新农合市级统筹和大病保险，力争新农合参合率稳定在98%以上。启动第二批县级医院综合改革，巩固完善基本药物制度。

　　强化公共服务。实施教育提升工程，加快学前教育普惠发展，推进义务教育均衡发展，促进普通高中教育优质发展。办好荆楚理工学院职业技术学院，加强荆门技师学院建设。支持荆楚理工学院加快发展，确保顺利通过教育部本科教学工作合格评估。统筹配置卫生计生资源，进一步落实计划生育国策，建立“便民、惠民、高效”的服务体系，创建国家卫生城市。力争市一医南城区医院、市精神卫生中心和市中医医院住院大楼建成投入使用。开工建设市博物馆新馆、荆楚文化广场、工人文化宫和“三个中心”等公共文化项目。开展全民阅读月系列活动，创建“农家书屋示范县（市）”。依法加强文物保护。组建文化体育产业集团，支持广电、报业和文体三大产业集团做大做强。弘扬科学精神，提高全民科学素养。促进文艺精品创作，发展哲学社会科学，出版《荆门市志》。用好新媒体，弘扬主旋律。积极备战省十四运会，广泛开展全民健身活动。

　　创新社会治理。进一步扩大基层民主，做好第九届村民委员会换届工作。实施中心城区社区建设三年行动计划，完善网格化社会管理和数字化城市管理联系会议制度，进一步抓好“四管两中心”建设。持续推进“六五”普法，加强和改进信访工作，完善人民调解、行政调解、司法调解联动工作机制，推进社会稳定风险评估全覆盖。关爱农村留守儿童、妇女和老人，加强流动人口和特殊人群服务管理。深化平安荆门建设，创新立体化社会治安防控体系，依法严密防范和惩治各类违法犯罪行为。狠抓食品药品监管，确保食品药品安全。加强企业安全生产基础建设，健全应急处理机制，有效防范和遏制较大以上事故发生。依法管理民族宗教事务，继续做好援疆和援恩工作。抓好防震减灾，提升人防应急能力。认真开展第三次经济普查。力争国家安全、保密、对台、外事、侨务、统计、残疾人、档案、气象等工作再上新台阶。

　　加强全民国防教育，扎实推进国防动员和民兵预备役工作。落实优抚安置政策，深入开展双拥共建，巩固发展军政军民团结。

　　各位代表，民生是为政之本。今年我们将优先办好以下民生实事：

　　1.城镇新增就业45000人，组织城乡劳动者就业培训19000人，扶持创业5500人，带动就业22000人。

　　2.葛洲坝水泥厂老生产线关闭，开展机动车尾气检测等污染防治，实施竹皮河汇合口浏河段和海慧沟段截污改造、生态补水等工程。

　　3.开工建设保障房、棚户区改造住房16082套（户），基本建成11600套（户），分配入住7800套（户）；新增发放廉租房租赁补贴550户；新建12个省级示范宜居村庄，完成5000户农村危房改造。

　　4.完善农村居民大病保障制度，对参合农民自付部分超过8000元以上合规费用进行二次补偿。

　　5.建设社区平价蔬菜直销店40家，方便城市居民生活。

　　6.对市特殊教育学校学生实行“四免政策”；为困难群众、弱势群体和农民工提供法律援助；开展残疾人实用技术培训3200名、城镇残疾人职业能力培训500名；免费为贫困残疾人适配1000件辅助器具。

　　7.新建10所留守儿童服务站，新建、改扩建公办幼儿园20所。

　　8.中心城区新增10处免费公共无线上网服务区，完成文化惠民演出110场。

　　9.新建50个城市社区居家养老服务中心、100个农村老年人互助照料中心，养老机构新增床位1000张，实施80岁以上老人高龄补贴制度。

　　10.对全市22个省市级重点老区、贫困村，实施扶贫整村推进工作，每个村整合资金不少于70万元。

　　各位代表，全面完成今年的目标任务，各级政府责任重大。我们将进一步加大政府自身改革和建设力度，使政府治理更加有效、行为更加规范、服务更加到位、运转更加顺畅。

　　切实在转变职能上有新突破。坚持“政府的归政府、市场的归市场、社会的归社会”，充分履行公共服务、市场监管、社会管理、环境保护的职责。以“五减四制三集中”为主要内容，深入推进行政审批制度改革，实现服务优质、环境优化。创新行政管理方式，推进政务信息化，加强发展战略、规划、政策的制定和实施。健全政府工作目标体系、考核办法、奖惩机制，提高行政效能和服务质量。

　　切实在改进作风上有新成效。认真落实中央八项规定、省委六条意见和市委实施细则，促进政风行风进一步好转。积极开展第二批党的群众路线教育实践活动，强化为基层服务、深化为企业服务、细化为群众服务。深入开展“效能风暴”行动，深化电视问政，加强机关作风明察暗访、电子监察、问责追查，推进作风建设常态化。巩固“三短一简”成果，坚决克服形式主义、官僚主义、享乐主义和奢靡之风。

　　切实在依法行政上有新作为。大力推进法治政府、法治机关、法治社会一体建设，自觉接受人大法律监督和工作监督，主动接受政协民主监督，虚心接受舆论监督和群众监督，认真办理人大代表建议和政协委员提案。健全行政决策规则，规范决策程序。高水平制定政府规范性文件。

　　严格规范行政执法，完善行政复议机制。进一步落实行政首长出庭应诉各项规定。大力推进政务公开，提高政府公信力。

　　切实在廉洁从政上有新形象。全面落实党风廉政建设责任制，加大从源头上预防和治理腐败力度。认真落实《党政机关厉行节约反对浪费条例》和《党政机关国内公务接待管理规定》，严格财务预算、核准和审计制度，从严控制“三公”经费支出。加强对重点资金、重大项目和领导干部经济责任审计，强化行政监察和审计监督。坚决纠正部门和行业的不正之风，继续加大查办案件工作力度，着力建设为民务实清廉政府。

　　各位代表，全面深化改革的号角已经吹响，加快转型升级的征程全面开启。让我们紧密团结在以习近平同志为总书记的党中央周围，在省委、省政府和市委的正确领导下，解放思想，同心同德，奋发有为，为加快建设实力文化生态幸福新荆门而努力奋斗！
